# Multi-Omics Feature Selection to Identify Biomarkers for Hepatocellular Carcinoma

**DOI:** 10.3390/metabo15090575

**Published:** 2025-08-28

**Authors:** Rency S. Varghese, Xinran Zhang, Sarada Giridharan, Muhammad Salman Sajid, Md Mamunur Rashid, Alexander Kroemer, Habtom W. Ressom

**Affiliations:** 1 Department of Oncology, Lombardi Comprehensive Cancer Center, Georgetown University Medical Center, Washington, DC 20057, USA; rsv4@georgetown.edu (R.S.V.); xz562@georgetown.edu (X.Z.); sg1817@georgetown.edu (S.G.); ms4868@georgetown.edu (M.S.S.); mr1785@georgetown.edu (M.M.R.); 2 MedStar Georgetown Transplant Institute, MedStar Georgetown University Hospital and the Center for Translational Transplant Medicine, Georgetown University Medical Center, Washington, DC 20057, USA; alexander.kroemer@gunet.georgetown.edu

**Keywords:** multi-omics approaches, liver cancer, LC-MS/MS, machine learning, deep learning, feature selection

## Abstract

Introduction: Hepatocellular carcinoma (HCC), the most prevalent form of liver cancer, ranks as the third leading cause of mortality globally. Patients diagnosed with HCC exhibit a dismal prognosis mostly due to the emergence of symptoms in the advanced stages of the disease. Moreover, conventional biomarkers demonstrate insufficient efficacy in the early detection of HCC, hence highlighting the need for the identification of novel and more effective biomarkers. Methods: In this paper, we investigate methods for integration of multi-omics data we generated by both untargeted and targeted mass spectrometric analysis of serum samples from HCC cases and patients with liver cirrhosis. Specifically, the performances of several feature selection methods are evaluated on their abilities to identify a panel of multi-omics features that distinguish HCC cases from cirrhotic controls. Results: The integrative analysis identified key molecules associated with liver including such as leucine and isoleucine as well as SERPINA1, which is involved in LXR/RXR Activation and Acute Response signaling. A new method that uses recursive feature selection in conjunction with a transformer-based deep learning model as an estimator led to more promising results compared to other deep learning methods that perform disease classification and feature selection sequentially. Conclusions: The findings in this study reinforce the importance of adapting or extending deep learning models to support robust feature selection, especially for integration of multi-omics data with limited sample size to avoid the risk of overfitting and the need for evaluation of the multi-omics features discovered in this study via blood samples from a larger and independent cohort to identify robust biomarkers for HCC.

## 1. Introduction

Hepatocellular carcinoma (HCC) ranks among the most common cancers globally and is one of the leading causes of cancer-related deaths [1]. Liver cancer incidence is expected to exceed one million new cases in 2025, making it one of the leading causes of cancer mortality across multiple developed nations, including the United States. Liver cirrhosis (CIRR) is the primary risk factor for HCC, [2,3,4] with approximately 90% of HCC cases arising in individuals with long-standing liver damage [5]. However, the progression from cirrhosis to HCC often remains asymptomatic [6,7], leading to delayed diagnosis when curative treatment options are limited [8]. Current diagnostic tools have significant limitations, including low sensitivity and specificity, particularly for early-stage HCC [9]. Various potential protein biomarkers are reported to enhance early diagnosis, prognosis, and therapeutic strategies in HCC management [4]. Although new candidate biomarkers (e.g., AFP-L3, DCP, GP-73) may improve HCC detection when used in conjunction with AFP, the sensitivity and specificity remain unsatisfactory [10,11]. Thus, more sensitive and potent biomarkers are highly desired for the early detection of HCC in patients with liver cirrhosis.

The integration of multi-omics data has become a powerful strategy for identifying robust biomarkers for complex diseases such as HCC. Combining multi-omics data from studies such as proteomics, metabolomics, lipidomics, glycoproteomics, etc., provides a high-resolution molecular portrait of cellular states and disease phenotypes. Each of these omics layers captures distinct yet complementary biochemical information. While proteomics reveals the functional protein landscape, glycomics and glycoproteomics shed light on post-translational modifications, metabolomics and lipidomics offer snapshots of cellular metabolism, and endogenous peptides reflect proteolytic processing and signaling dynamics. Recent advances in computational frameworks have made it feasible to harmonize such heterogeneous data, though challenges in normalization, dimensionality, and interpretability remain significant obstacles [12,13,14].

Multi-omics integration enables a deeper comprehension of the roles and interactions of different molecular entities in biological processes and disease mechanisms. Additionally, combining data from several omics layers enables cross-validation, which raises the dependability of the biomarkers found by confirming results from one layer [14].

Multi-omics data integration strategies often use knowledge-driven or data-driven methods. The former uses results from independent analysis of individual omics data to find molecular characteristics, which are then mapped into knowledge databases to find molecular relationships and pathways. The quality and extent of the databases limit this technique, making it suitable for omics data types and well-studied disorders. The latter integrates at the data level to find correlations and common patterns among omics layers [15].

Multiple tools and platforms have been developed for multi-omics integration at various levels. For example, xMWAS, 3Omics, and OmicsNet use correlation-based networks to integrate various omics datasets [16,17,18]. Galaxy and KNIME provide tools and workflows for specific multi-omics integration tasks. Galaxy provides a web-based interface with pre-configured workflows, making it accessible for users performing reproducible bioinformatics tasks [19,20,21]. KNIME offers a node-based environment for complex data integration, machine learning, and custom analytics across diverse omics and clinical datasets [22]. Other tools such as MixOmics [23], iClusterPlus [24], and Multi-Omics Factor Analysis (MOFA) [25] provide a range of methods for integrative analysis of multi-omics datasets. Pathview and SPIA integrate multi-omics data at a pathway level to understand how different biological pathways are affected [26,27,28]. JIVE analyzes multiple high-dimensional data types to identify joint and individual variations [29]. Non-negative Matrix Factorization (NMF) and its extensions, such as intNMF and nNMF, have been utilized for integrative clustering in disease subtype classification and for analyzing interconnected datasets in an unsupervised manner, respectively [30,31,32]. Another set of approaches that leverage graph convolutional networks for cancer subtype classification and biomarker identification include MoGCN and MOGONET [32,33]. On the same lines, DeepLIFT, employs meta-learning for interpretable multi-omics analysis and pathway enrichment [34]. DeePathNet and Pathformer are transformer-based deep learning models that integrate multi-omics data and pathways for disease prediction and identifying deregulation of pathways [35,36].

In this study, we examined data from two (untargeted and targeted) multi-omics studies conducted by mass spectrometry to identify key molecules that differ between HCC vs. CIRR. Following significance analysis using Student’s *t*-test, we evaluated SelectKBest, support vector machine–recursive feature elimination (SVM-RFE), and Transformer–SVM for multi-omics feature selection. In addition, we built classifiers using random forest (RF), the multi-omics early integration framework (MOINER) [37], and Multi-Omics Graph cOnvolutional NETworks (MOGONET). The features are then ranked according to their feature importance or the SHapley Additive exPlanation (SHAP) values. Pathway analysis revealed insights into the molecules and their interactions at the disease pathway level.

## 2. Materials and Methods

### 2.1. Untargeted Multi-Omics Studies

**Overview.** Serum samples from 20 HCC cases and 20 liver cirrhosis patients recruited at MedStar Georgetown University Hospital were analyzed in untargeted multi-omics studies using liquid chromatography coupled with tandem mass spectrometry (LC-MS/MS). All participants filled out HIPAA authorization forms and provided their informed consent. The characteristics of the subjects whose serum samples were studied using multi-omics techniques are shown in Table 1. Every HCC patient in this study had a diagnosis of liver cirrhosis. Well-established criteria for diagnostic imaging and/or histology were used to diagnose cases with HCC. The tumor–node–metastasis (TNM) classification system was used to determine the clinical stages of HCC.

**Figure 1:**
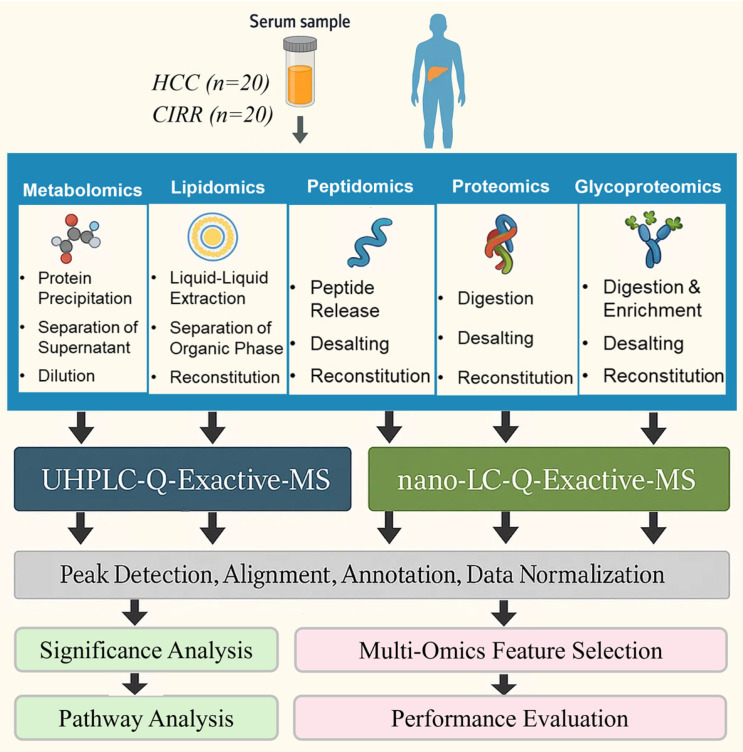
Workflow for integrative analysis of data acquired by untargeted multi-omics studies.

Figure 1 depicts our workflow for multi-omics integrative analysis. As shown in the figure, LC-MS/MS data from each omics study were separately processed to detect peaks, align peaks, annotate analytes, and normalize peak intensities. In order to identify multi-omics features and disease pathways linked to HCC, the processed data were merged for further analysis. In the following, we describe the sample preparation, data acquisition, and data processing steps we followed for each omics study as well as subsequent analyses including integration of multi-omics data and pathway analysis.

**Metabolomics and Lipidomics**: For metabolomics data acquisition, 50 μL serum aliquot was mixed with 150 μL chilled methanol, vortexed, and centrifuged, and the supernatant was collected. It was then mixed with water containing internal standards (debrisoquine sulfate for positive mode, 4-nitrobenzoic acid for negative mode). A 5 μL volume was injected into the Vanquish UHPLC-Q-Exactive-MS system. QC samples, prepared by pooling and diluting serum with internal standard-containing water, were used for system conditioning and injected after every 10 samples to monitor analytical consistency. Chromatographic separation was performed on an ACQUITY UPLC BEH C18 column, using HESI in both positive and negative ionization modes [38].

For lipidomics profiling, serum (50 μL) was mixed with 25 μL PC (16:0/18:1)-d31 (positive mode internal standard), 25 μL arachidonic acid-d8 (negative mode internal standard), and 50 μL 0.1 M NaCl, followed by extraction with 250 μL chloroform: methanol (1:2, *v*/*v*). QC samples, prepared by pooling equal volumes of reconstituted samples and processed identically, were used to assess instrument consistency. Chromatographic separation employed an ACE Excel 2 Super C18 column, and LC-MS/MS data were collected using the same system as for metabolomics [38].

Raw Q-Exactive-MS data from metabolomics and lipidomics studies were processed using Compound Discoverer 3.1 (Thermo Fisher Scientific, Waltham, MA, USA) for peak alignment, detection, and identification, as both studies used identical MS conditions. Data were normalized to internal standards: debrisoquine sulfate and 4-nitrobenzoic acid for metabolomics, and PC (16:0/18:1)-d31 and arachidonic acid-d8 for lipidomics. Positive and negative mode data were then combined. Putative metabolite IDs were assigned based on mass adducts ([M+H]^+^, [M+Na]^+^, [M+NH_4_]^+^, [M−H]^−^, etc.) and MS/MS fragmentation using tools and databases including MetaboQuest, Compound Discoverer, LipidSearch, HMDB, and METLIN.

**Peptidomics and Proteomics**. For peptidomics profiling, serum (40 µL) was mixed with 250 µL 1% TFA, heated at 98 °C for 10 min, centrifuged (14,000× *g*, 20 min, 4 °C), washed twice with 1% TFA, and centrifuged again. Extracted peptides were desalted, resuspended in 2% ACN/0.1% FA, and quantified before nano-LC-MS/MS analysis. Positive mode data were acquired using a Dionex 3000 UltiMate Nano LC coupled to a Q-Exactive mass spectrometer at 2.2 kV. Full MS scans (370–1850 m/z) were collected at 70,000 resolution; the top 10 ions underwent HCD MS2 (NCE 27.5). A 1 µg quantity of peptides was injected onto a C18 PepMap trap and RSLC column using a 145 min multistage gradient.

For proteomics data acquisition, depleted serum proteins were dissolved in 7 M urea/100 mM NH_4_HCO_3_, reduced with 50 mM DTT, and centrifuged (14,000× *g*, 14 min) using a 30 kDa spin filter. Proteins were alkylated with 55 mM IAA (20 min, dark), washed with 50 mM ABC, and digested overnight at 37 °C with trypsin (1:30, enzyme/protein). Peptides were desalted, resuspended in 2% ACN/0.1% FA, and quantified before nano-LC-MS/MS analysis. Data were acquired using a Dionex 3000 UltiMate Nano LC coupled to a Q-Exactive mass spectrometer. Full MS scans (370–1850 m/z) were collected at 70,000 resolution; the top 10 ions underwent HCD MS2 (NCE 27.5) at 17,500 resolution with 20 ms dynamic exclusion. A 1 µg quantity of peptides was injected onto a C18 PepMap trap and RSLC column with a 145 min multistage gradient.

Label-free quantification (LFQ) of the peptidomics and proteomics data as well as peptide identification were performed using Proteome Discoverer 3.0 software (Thermo Scientific, USA) with a human database (July 2023) using the Sequest HT search algorithm. The processing workflow involved the mass recalibration node, Minora Feature Detector, standard spectrum selector, Sequest HT, and Percolator nodes. Precursor mass tolerance was set at 10 ppm, and fragment mass tolerance was set to 0.02 Da with no specific enzyme for endogenous peptidomics and trypsin for proteomics with one missed cleavage.

**Glycoproteomics**: Digested peptides were dried and reconstituted in 500 µL loading buffer (ACN/H_2_O/TFA, 92/7/1), then mixed with 500 µg equilibrated HILIC sorbent. After 10 min incubation, sorbent-bound glycopeptides were washed twice with loading buffer and once with washing buffer II (ACN/H_2_O/H_3_PO_4_, 85/14.5/0.5). Glycopeptides were eluted with 50 µL elution buffer (ACN/H_2_O/TFA, 30/69.9/0.1), lyophilized, and resuspended in 2% ACN, 0.1% FA for quantification and nano-LC-MS/MS analysis. MS data were acquired in positive mode using a Dionex 3000 UltiMate Nano LC coupled to a Q-Exactive mass spectrometer (Thermo Scientific) at 2.4 kV. Full MS scans (280–1800 m/z) were recorded at 70,000 resolution, with the top 10 ions selected for HCD MS2 fragmentation (NCE 28, 30, 32) at 17,500 resolution and a dynamic exclusion of 30 ms.

Glycoproteomics data were analyzed using PD 3.0 with Byonic, searching a human database containing 182 N-glycans and 70 O-glycans. LFQ and glycopeptide identification were performed with 10 ppm precursor and 0.02 Da fragment mass tolerance using trypsin. Carbamidomethylation was set as a static modification, with oxidized methionine as a variable modification. Manual validation was based on oxonium ions: m/z 204.09, 292.10, 274.09, 366.14, and 512.20 for N-linked glycans, and m/z 126.05, 138.05, 144.06, 168.06, 186.08, or 204.08 for O-linked glycans.

**Statistical Significance and Pathway Analysis**. Following statistical analysis by Student’s *t*-test, pathway analysis is performed using Ingenuity Pathway Analysis (IPA, QIAGEN Inc., Germantown, MD, USA) based on the significant multi-omics features [39]. Top canonical pathways enriched with the significant molecules are identified. Key genes associated with liver disease are identified through Machine Learning Disease Pathways in IPA. Upstream regulator analysis identifies molecules that are upstream regulators and their targets that are significant in our study.

**Multi-Omics Feature Selection**. We combined the multi-omics data to select the most relevant panel of multi-omics features. Features missing in 30% or more of the samples were removed prior to creating a data matrix containing abundance values of all multi-omics features. Following significant analysis using Student’s *t*-test, integrative analysis of the combined multi-omics data was performed by SelectKBest, support vector machine–recursive feature elimination (SVM-RFE), Transformer–RFE, random forest (RF), MOINER, and MOGONET [37]. While the first three rank the multi-omics features directly, the last three build first disease classification models, followed by ranking the features based on their significance to the classification task. This is accomplished by building a logistic regression model for classification and calculating either feature importance scores or SHAP values to rank the features [40].

SelectKBest is a filter-based feature selection method in scikit-learn that selects the top K features based on a univariate statistical test [41]. An F-score is used to rank the features according to their relationship with the output variable. Then, the K best features with the highest scores are selected as the feature subset. The method reduces overfitting by removing irrelevant features.

Support vector machine–recursive feature elimination (SVM-RFE) is a widely used supervised feature selection technique that aims to select features by recursively removing the least important ones based on model performance. In SVM-RFE, the SVM is trained as an estimator first trained on the entire feature set [42]. The magnitudes of the weight vector serve as feature-importance scores, and the least important features are systematically removed. This process is repeated recursively on the remaining set until a pre-specified number of features is selected.

Transformer–recursive feature elimination (Transformer-RFE) is a new method we implemented inspired by SVM-RFE. The method replaces the SVM with a lightweight cross-attention transformer. The transformer is first trained on the full multi-omics dataset; SHAP values of the input features provide feature importance scores. After each training run the feature with the lowest score is dropped, and the model is refit on the trimmed feature set. The loop continues until a pre-specified number of features remains.

Random forest (RF) is an ensemble-based supervised learning algorithm that constructs a collection of decision trees during training and aggregates their predictions to improve classification or regression performance. Each tree in the forest is trained on a bootstrapped subset of the data, and at each split within a tree, a random subset of features is considered, promoting diversity among trees [43]. This randomness helps reduce overfitting compared to a single decision tree and enhances generalization.

MOINER employs self-attention mechanism to capture the correlations of omics-features and uses them to perform disease classification. Information enhancement is performed through neighborhood aggregation and message passing in the sample similarity network (SSN), thereby encapsulating the information of the data. Vision transformer (ViT) is then used for conducting classification. Thus, the method embeds multi-omics profiles as images and leverages deep attention to integrate heterogeneous data. Chi-square scores are used to select top 500 features from each omics dataset prior to feature selection using the MOINER framework [37].

MOGONET first constructs an SSN for each omics type based on cosine similarity of feature profiles, then trains parallel graph convolutional networks (GCNs) to learn view-specific embeddings that are fused by the View Correlation Discovery Network (VCDN). By combining these omics-specific GCNs with VCDN, the model captures cross-omics correlations in label space. The final classification is performed using VCDN [44].

**Performance Evaluation**. The ability of the top five features selected by each of the six methods was evaluated by using them as a panel to build a logistic regression model to classify HCC vs. CIRR.

### 2.2. Targeted Multi-Omics Studies

**Figure 2:**
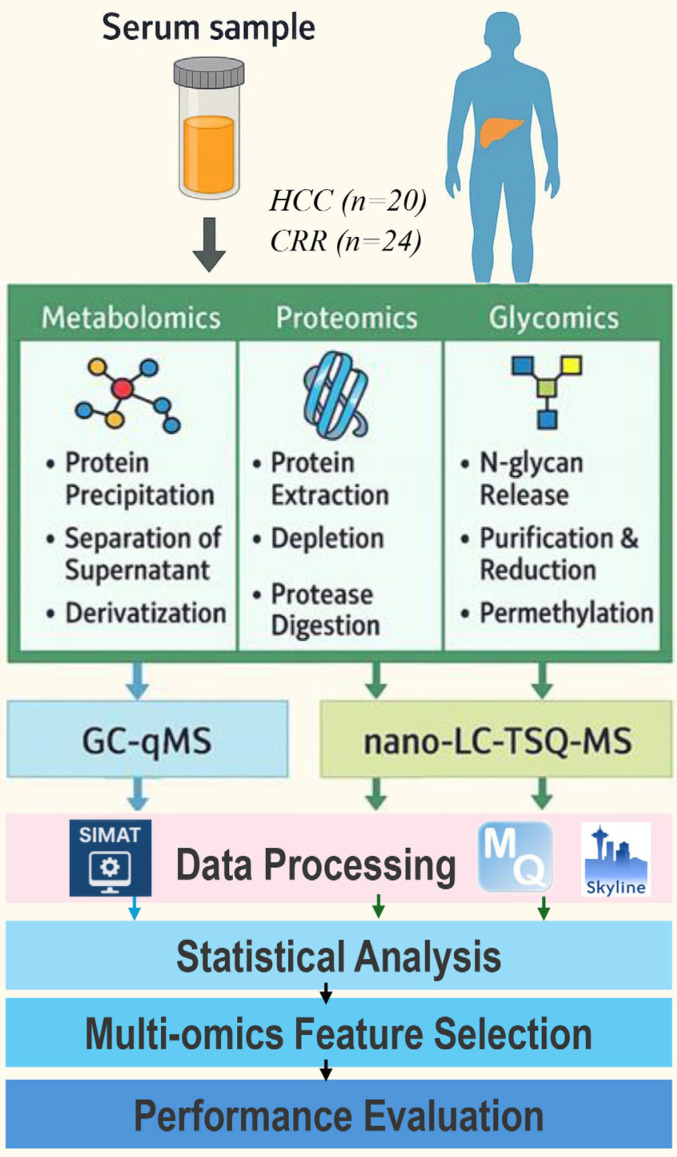
Workflow for targeted multi-omics studies.

**Overview**. Blood samples were analyzed from 44 subjects (20 HCC cases and 24 patients with liver cirrhosis) recruited from the hepatology clinics at MedStar Georgetown University Hospital. All participants filled out HIPAA authorization forms and provided their informed consent. Table 2 presents the characteristics of the 44 subjects. Figure 2 illustrates our workflow for targeted multi-omics studies. In the following, we describe the sample preparation, data acquisition, and data processing methods we used for each omics study and how the multi-omics data are integrated to identify a panel of multi-omics features that distinguishes HCC cases from cirrhotic controls.

**Metabolomics**: Fifty metabolites selected from a previous untargeted study were subjected for a targeted analysis in blood samples by selected ion monitoring (SIM) using an Agilent 7890A GC interfaced to a single quadrupole Agilent 5975C MSD (Agilent Technologies, Santa Clara, CA, USA). Plasma metabolites were extracted by adding 1 mL of working solution composed of acetonitrile, isopropanol, and water (3:3:2) containing isotope-labeled internal standards at a concentration of 1.25 μg/mL to 30 μL of plasma. The dried samples were derivatized prior to injection following a two-stage process of oximation followed by trimethylsilylation (TMS) [45].

The GC-qMS data acquired were processed using SIMAT [46]. Before peak detection, a smoothing filter is used. Then, the profiles of the quantifiers are utilized to locate and evaluate the reliability of the corresponding fragment peaks for each target. This is achieved by comparing the similarity scores of all candidate peaks with the target analyte using spectral matching and RI distance. Results from univariate statistical analysis have been previously reported in [45,47,48].

**Proteomics and Glycomics**: Prior to MRM scheduling of individual samples, a 1 μL aliquot of each sample was pooled and subjected to MRM experiment to refine the transition list. A 3 μL aliquot of the pooled sample was analyzed by LC-MRM-MS. Data independent acquisition mode was used for MRM experiment. Predefined precursor and transition ions were monitored to specifically select targeted peptides corresponding to each candidate protein with 10.0 sec chromatogram filter peak width. Targeted quantitative analysis of 101 selected proteins and 82 N-glycans in blood samples was performed by multiple reaction monitoring (MRM) using a Dionex 3000 Ultimate nano-LC system (Dionex Sunnyvale, CA, USA) interfaced to TSQ Vantage mass spectrometer (Thermo Scientific, San Jose, CA, USA). The targets were selected from our previous LC-MS-based untargeted proteomic and glycomic analyses and by text mining. The LC-MRM-MS data were analyzed using Skyline (version 2.5.0.6079) [49]. Peptide search results from Andromeda, to recognize the monitored transitions from LC-MRM-MS data [47,50].

**Multi-Omics Feature Selection and Performance Evaluation**. Following statistical analysis using Student’s *t*-test, integrative analysis of the combined multi-omics data was performed by SelectKBest, SVM-RFE, Transformer–RFE, RF, MOINER, and MOGONET to identify multi-omics features that distinguish HCC cases from patients with liver cirrhosis. The top five features selected by each of the six methods were then valuated by using them as a panel to build a logistic regression model to classify HCC vs. CIRR.

## 3. Results

### 3.1. Untargeted Multi-Omics Studies

**Statistical Significance and Pathway Analysis**. Table 3 summarizes the number of features identified in each omics data after data processing using Student’s *t*-test. As shown in the table, a large number of statistically significant features are identified by analysis of the proteomics data. In order to evaluate the performance of feature selection methods reasonably without being overly dominated by the proteomics features, we performed the subsequent analyses by excluding the proteomics features.

The significant features identified using Student’s *t*-test with FDR < 0.05 and the top 100 selected features using SelectKBest were used for pathway analysis. Each feature was annotated with either a KEGG ID, PubChem CID, HMDB ID, or UniProt ID. Canonical pathway analysis revealed significant pathways, molecules, upstream regulators, and networks that are altered in HCC vs. CIRR based on our multi-omics data. There was significant overlap between the pathways and the upstream regulators from the Student’s *t*-test and SelectKBest analyses.

**Figure 3:**
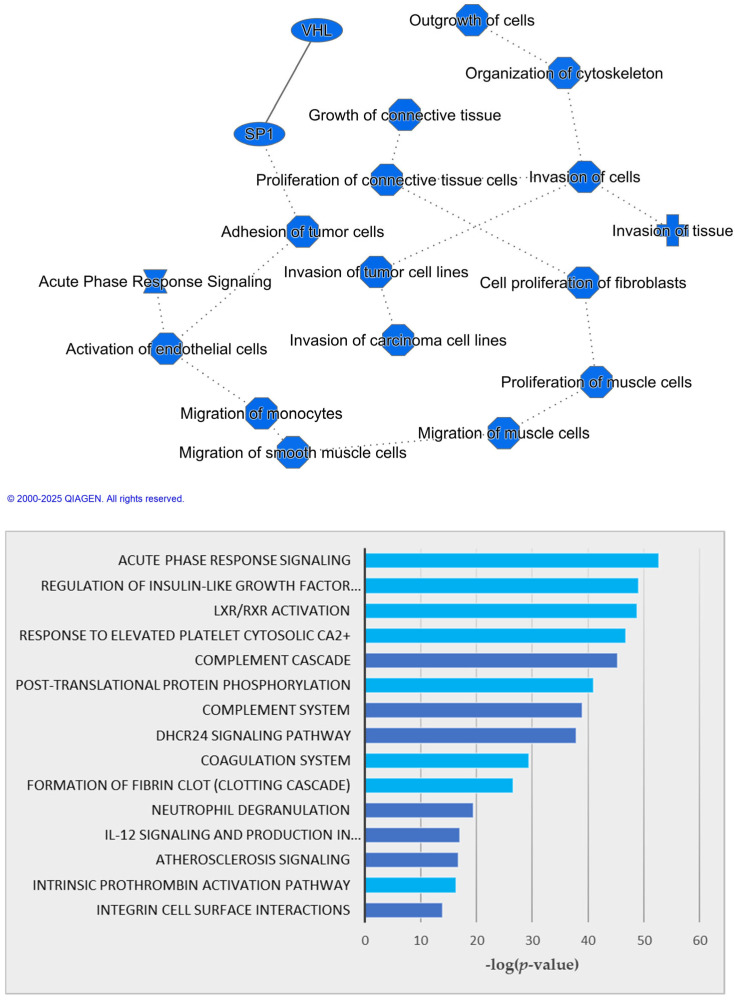
Graphical summary of the interactions between top molecules and pathways (**top**). The solid line represents a direct interaction whereas the dotted lines refer to inferred relationships. Top 15 canonical pathways with *p* < 0.05 using IPA analysis (**bottom**).

**Figure 4:**
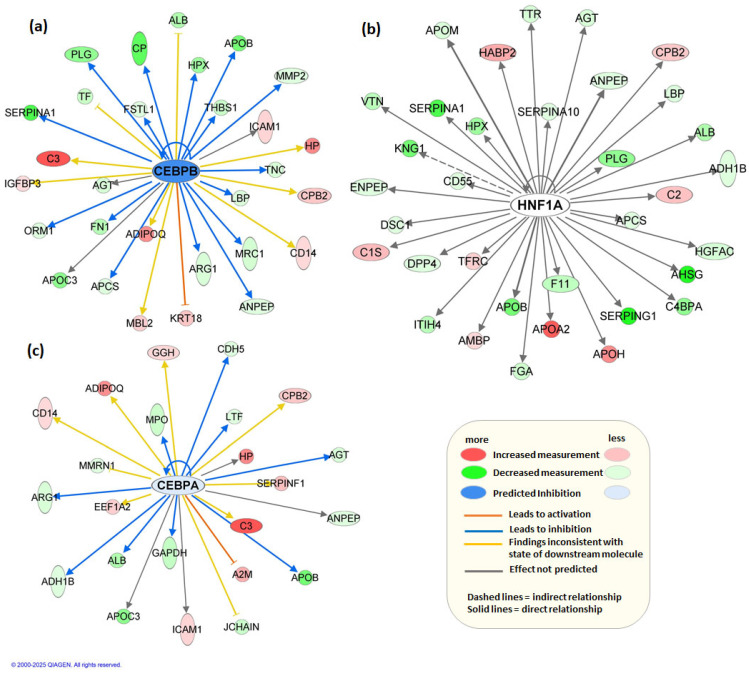
Upstream regulator network predicted by Ingenuity Pathway Analysis (IPA). The top three regulators are represented as nodes *CEBPB* (**a**), *HNF1A* (**b**), and *CEBPA* (**c**), with their activation state indicated by node color (blue = predicted inhibition, white = no prediction). Target molecules are shown as downstream nodes, and edges represent activation (orange lines) or inhibition (blue lines) relationships.

Figure 3 (top) shows the graphical summary of the regulators, pathways, diseases, and functions predicted based on the molecules obtained by combining the statistically significant features from each untargeted omics study. Figure 3 (bottom) shows the top canonical pathways enriched by the significant molecules with FDR < 0.05 from the integrated analysis including proteomics data. Figure 4 illustrates the upstream regulator analysis network, which predicts the molecules that are activating or inhibiting the observed expression changes.

Pathway analysis using the top 25 features selected by transformer models excluding proteomics features also revealed significant overlap (8 in the top 10) in the canonical pathways, as highlighted in blue in Figure 3 (bottom). The pathways identified with and without the proteomics data confirms the potential interactions between the molecules represented by the multi-omics features.

**Figure 5:**
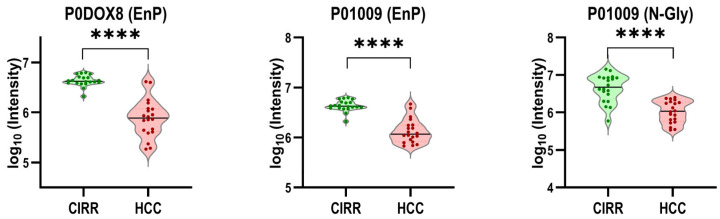
Violin plots for three examples of top features selected by integrative analysis (**** denotes *p*-value < 0.0001).

**Multi-Omics Feature Selection**. The data from the multi-omics studies are introduced to six feature selection methods. Table 4 shows the top five features ranked by each method. Key identifiers (protein IDs, metabolite/lipid names) are listed under each method. The features span multiple omics layers, including proteins, metabolites, lipids, endogenous peptides (represented by their master protein) and glycoproteins. Overlapping features across methods highlight potential biomarkers. Figure 5 depicts the violin plots of the two most frequently selected features—P0DOX8 (EnP) and P01009 (EnP and N-Gly).

**Performance Evaluation**. The predictive capacity of the top five features was evaluated using a logistic regression classifier with five-fold cross-validation. As shown in Table 4, SelectKBest and RF achieved 100% classification accuracy, indicating highly consistent feature selection. The remaining methods also performed strongly. MOINER showed comparatively lower classification accuracy, possibly due to inadequate sample size. Also, as shown Table 4, each feature selection method achieved very high predictive performance based on receiver operating characteristic (ROC) and their corresponding area under the curve (AUC) values. The table shows the average AUC values over a five-fold cross-validation of a logistic regression classifier trained using the top five features selected by each method.

### 3.2. Targeted Multi-Omics Studies

**Statistical Significance Analysis**. Table 5 summarizes the number of significant features identified by Student’s *t*-test from 101 proteins, 53 metabolites, and 82 glycans.

**Multi-Omics Feature Selection**. The data from the targeted omics studies are introduced to the same feature selection methods as in the untargeted studies to select the top five features. Table 6 illustrates both shared and unique features identified by different feature selection techniques.

**Figure 6:**
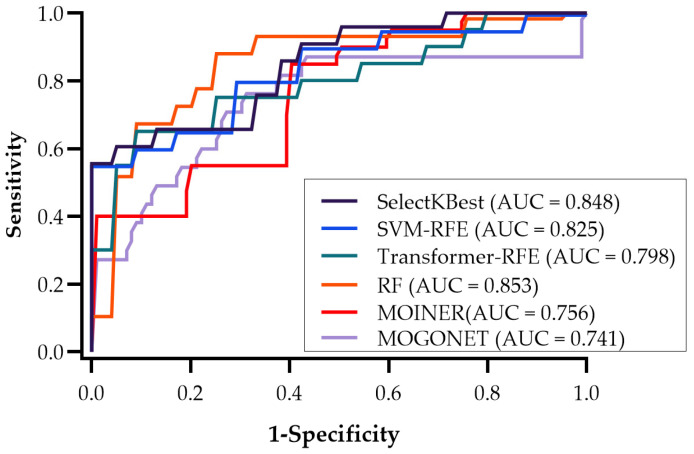
Performance evaluation of the top five multi-omics features selected by each feature selection method. AUC: area under the curve of recursive operating characteristics (ROC).

**Performance Evaluation**. Figure 6 depicts the ROC curves and their corresponding AUC values calculated based on a logistic regression classifier using the top five features selected by each method. As shown in the figure, RF yielded the highest AUC (0.853). SelectKBest and SVM-RFE performed comparably well, with AUCs above 0.8. Transformer–RFE, MOINER, and MOGONET yielded promising results but slightly less classification accuracy and AUC values than the more traditional methods in this dataset. Overall, these results support the growing recognition that machine/deep learning-based integrative frameworks can be effective at identifying biomarkers in complex, high-dimensional multi-omics datasets. This has implications for future biomarker discovery pipelines, particularly in diseases such as cancer where biological processes are inherently multifactorial.

## 4. Discussion

The network in Figure 3 (top) represents the most significant predicted entities by IPA based on statistically significantly altered features in the untargeted multi-omics data. These features are mainly involved in Endothelial Cell Activation and Acute Phase Response, Tumor Cell Adhesion and SP1 Regulation, Fibroblast Proliferation and Connective Tissue Dynamics, Cell Invasion and Cytoskeletal Organization, and Monocyte and Muscle Cell Migration. The network indicates that a decrease in the acute phase response signaling leads to reduced activation of endothelial cells. The decrease in SP1 leads to reduced adhesion of tumor cells, highlighting the role of SP1 as a transcription factor in regulating genes involved in cell adhesion.

Among the multi-omics features used for pathway analysis, 44 molecules were associated with regulating insulin-like growth factor (IGF) transport and uptake by IGF-binding proteins (IGFBPs) and 43 molecules found associated with activating liver X receptors (LXR) or retinoid X receptors (RXR). Acute phase response is a rapid inflammatory response that provides protection against microorganisms using non-specific defense mechanisms. LXR/RXR is involved in the regulation of lipid metabolism, inflammation, and cholesterol to bile acid catabolism. Genes regulated by LXR include the ATP-binding cassette transporter A1 (ABCA1), which effluxes cholesterol from extrahepatic cells; the sterol regulatory element binding protein-1c (SREBP-1c); AIM; and LPL. These pathways were reported in our previous study conducted by analysis of sera and tissues from HCC cases and cirrhotic patients [51]. In the complement cascade, a panel of soluble molecules rapidly and effectively senses a danger or damage and triggers reactions to provide a response that discriminates among foreign intruders, cellular debris, and healthy and altered host cells [52].

Concurrently, upstream regulator analysis revealed top regulators, *CEBPB*, *HNF1A*, and *CEBPA* as potential key drivers of the observed molecular changes. These are important transcription factors with diverse roles in liver development, function, and regeneration, as well as in the pathogenesis of HCC [53,54]. Many of the genes influenced by the decrease in *CEBPB*, such as *CD14*, *MBL2*, *ICAM1*, and *HP*, are known to play roles in the inflammatory response (Figure 4a). The increase in these genes suggests a potential enhancement in inflammation-related processes. Genes like *ALB*, *APOB*, and *APOC3* are involved in protein and lipid metabolism. The downregulation of these genes implies a suppression of metabolic pathways and transport functions. Several genes such as *CP*, *HP*, and *ORM1* are involved in the acute-phase response, indicating that a decrease in *CEBPB* can influence systemic responses to injury, infection, or other stress factors. In the *HNF1A* network, the decrease of *APOB* and increase of *APOA2* and *APOH* indicate significant regulation in lipid transport and metabolism (Figure 4b). The roles of these apolipoproteins in lipid binding, transport, and metabolism underscore the importance of lipid metabolism in the given network. The regulation of genes such as *ALB* (albumin), *AHSG* (alpha-2-HS-glycoprotein), *KNG1* (kininogen 1), and *HPX* (hemopexin) highlights the importance of liver function. Many of these genes are associated with plasma proteins synthesized by the liver. The network’s influence on *ADH1B* (alcohol dehydrogenase), *PLG* (plasminogen), and *SERPINA1* (alpha-1-antitrypsin) indicates metabolic regulation, including processes like alcohol metabolism, fibrinolysis, and protease inhibition. The genes affiliated with inflammation, such as *LBP* (lipopolysaccharide-binding protein), *SERPINA10* (protease inhibitor), and *CD55* (complement decay-accelerating factor), demonstrate inflammatory response, crucial for immune system regulation and pathogen defense. The downregulation of *CEBPA* leads to an increase in proteins associated with the immune system and inflammatory response, such as *A2M*, *C3*, *CD14*, *CPB2*, and *ICAM1* (Figure 4c). These genes are critical to the activation and regulation of immune responses, indicating a significant shift towards inflammation and immune defense mechanisms when *CEBPA* is decreased. The network shows that the decrease in *CEBPA* impacts several genes involved in metabolism such as *ADH1B*, *ALB*, *ARG1*, *GAPDH*, and *APOC3*. These genes play essential roles in the body’s metabolic pathways, involving lipid metabolism, energy production, and carbohydrate metabolism. Several genes related to lipid transport and metabolism are affected by the downregulation of *CEBPA*, including *A2M*, *APOB*, and *APOC3*. These genes are essential for lipid binding and transport, highlighting a potential disruption in lipid homeostasis when *CEBPA* is decreased. Predicted activation or inhibition of these regulators was supported by consistent directional effects on downstream targets, providing insights into the regulatory mechanisms underlying in HCC.

Machine learning disease pathways from IPA identified key genes that are associated with liver diseases from our data. This analysis selected 27 genes associated with the multi-omics data. Specifically, *C3* and *TF* were identified in peptidomics, proteomics, and N-linked glycoproteomics data as significantly altered in HCC vs. CIRR. *C3* is known to be upregulated by *STAT3* and is upregulated in our analysis and therefore *STAT3* is predicted to be activated in the causal network analysis. Overexpression and constitutive activation of *STAT3* have been frequently found in HCC and associated with poor prognosis. Ample evidence has shown that *STAT3* plays pivotal roles in the initiation, progression, metastasis, and immune suppression of HCC [55].

Multi-omics feature selection by six methods identified key molecules. For example, SelectKBest identified P80748, P0DOX8, P01009 (N-Gly), P01009 (EnP), and P0DOX5 as its top five features selected from the untargeted multi-omics data. Each of these features was also selected by at least one of the remaining methods. In particular, P01009 was selected as one of the top five features by all feature selection methods. Overexpression of P01009 (SERPINA1) in HCC vs. healthy controls has been reported. We previously reported SERPINA1 as a potential candidate in our proteomics study [56]. The candidate belongs to the LXR/RXR Activation pathway that is involved in regulating cholesterol and fatty acid metabolism.

P02656 was selected by Transformer–RFE and MOGONET, whereas heptacosanoic acid was selected by Transformer–RFE and MOINER. Heptacosanoic acid is a long-chain saturated fatty acid that falls within a metabolic class of fatty acids and lipid metabolism; it is recognized as central to HCC development and progression [57]. P80748 was selected by SelectKBest, SVM-RFE, and RF. Previous serum proteomics analyses have identified altered levels of IGLV3-21 (P80748) in HCC patients compared to healthy controls, suggesting its potential role as a biomarker. These results demonstrate that while overlapping features are selected, some feature selection methods tend to pick up unique and potentially complementary biomarker candidates. This reinforces the value of using diverse selection strategies in multi-omics studies to ensure both reproducibility and biological discovery.

Multi-omics features selected from the targeted multi-omics data by more than one method included leucine, isoleucine, O75636, P03952, Q6EMKA, P05156, and P01876. While leucine was selected by RF, MOINER, and MOGONET, isoleucine was selected by the former two. Branched-chain amino acids (BCAAs), i.e., valine, leucine, and isoleucine, have been reported to have connections with several types of cancer including HCC. They have also been connected to other liver diseases such as cirrhosis [58].

## 5. Conclusions

Multi-omics data acquired by analysis of blood samples from hepatocellular carcinoma (HCC) cases and patients with liver cirrhosis are analyzed to identify candidate biomarkers. Specifically, data from untargeted and targeted multi-omics studies acquired by mass spectrometry are analyzed to select a panel of multi-omics features that distinguishes HCC cases from cirrhotic controls. The untargeted multi-omics studies include metabolomics, lipidomics, peptidomics, proteomics, and glycoproteomics data, whereas the targeted studies consist of metabolomics, proteomics, and glycomics data. Prior to multi-omics feature selection by using various feature selection methods, we performed statistical significance analysis of all features detected in each omics study. We found much higher number of statistically significant and more correlated features in the untargeted proteomics study compared to the other omics studies. In order to reasonably evaluate the performance of the feature selection methods without being overly dominated by the features from the untargeted proteomics study, we performed the subsequent multi-omics feature section by setting aside the untargeted proteomics study for future integrative analysis. Thus, future work will focus on investigating new methods that allow for identification of seemingly uncorrelated multi-omics features from all multi-omics studies including the untargeted proteomics data to select a parsimonious panel of biomarker candidates. Furthermore, biomarker candidates discovered in this study will be evaluated via blood samples from a larger and independent cohort to identify robust biomarkers for HCC.

While deep learning methods such as MOINER and MOGONET typically demonstrate high classification performance, they are prone to overfitting especially when the sample size is small. In addition, these methods are not intrinsically structured to perform feature selection during model training. Instead, feature relevance is inferred post hoc using techniques such as feature importance and SHapley Additive exPlanations (SHAP) values, which analyze the contribution of features after a classifier has been trained. This contrasts with other approaches, such as recursive feature elimination (RFE), which iteratively remove or select features by retraining the classifier multiple times to evaluate the impact of each feature subset. Applying such recursive retraining strategies to MOINER or MOGONET would be computationally intensive due to the high complexity and resource requirements of such deep learning models.

We performed preliminary investigation on the use of RFE in conjunction with a transformer-based deep learning model as an estimator. The method led to more promising results compared to other deep learning methods that perform disease classification and feature ranking sequentially. These results reinforce the importance of adapting or extending deep learning models to support robust feature selection, especially for multi-omics data with limited sample size to avoid the risk of overfitting.

## Figures and Tables

**Table 1:** Characteristics of the patients whose serum samples were analyzed by untargeted studies. Characteristics with statistically significantly different values (*p* < 0.05) between the two patient groups are marked with asterisk.

		HCC (*n* = 20)	CIRR (*n*= 20)	*p*-Value
Age	Mean (SD)	59 (6)	57 (6)	0.487
Sex	Male	60%	65%	1
Race	AA	50%	40%	0.5231
	EA	50%	60%	
HCV Serology	HCV Ab+	80%	75%	0.6948
HBV Serology	anti HBC+	45%	40%	0.7431
	HBs Ag+	5%	0	1
Smoking	Current	25%	25%	1
	Former	55%	50%	
Alcohol	Current	25%	20%	0.6851
	Former	55%	60%	
MELD *	Median (IQR)	10.5 (5.2)	13.5 (9.2)	0.0475
AFP	Median (IQR)	29.1 (60.8)	7 (35.1)	0.113
HCC Stage	Stage I	30%		
	Stage II	65%		
	Stage III	5%		

**Table 2:** Characteristics of the patients whose serum samples were analyzed by targeted studies. Characteristics with statistically significantly different values (*p* < 0.05) between the two patient groups are marked with asterisk.

		HCC (*n* = 20)	CIRR (*n* = 24)	*p*-Value
Age	Mean (SD)	59.7 (6)	57.8 (7)	0.346
Sex	Male	80%	79%	1
Race *	AA	35%	17%	0.045
	EA	45%	70%	
HCV Serology	HCV Ab+	70%	42%	0.15
HBV Serology	anti HBC+	60%	21%	0.18
	HBs Ag+	10%	0	0.386
Smoking	Current	15%	22%	0.862
	Former	50%	48%	1
Alcohol	Current	20%	13%	0.84
	Former	50%	57%	0.904
MELD *	Median (IQR)	10 (4.3)	14.5 (6.3)	0.0004
AFP	Median (IQR)	29.1 (74.3)	4.2 (7.3)	0.0438
HCC Stage	Stage I	60%		
	Stage II	40%		

**Table 3:** Number of significant features found from each omics dataset using *t*-test.

Omics Study	No. of Features Detected	No. of Features (*p* < 0.05)	No. of Features (FDR < 0.05)
Metabolomics (Met)	7174	345	-
Lipidomics (Lip)	2252	397	117
Endogenous Peptidomics (EnP)	2355	438	70
N-linked Glycoproteomics (N-Gly)	750	164	49
O-linked Glycoproteomics (O-Gly)	244	74	67
Proteomics (Prot)	3530	1339	975

**Table 4:** Top five features selected from the untargeted multi-omics studies using SelectKBest, SVM-RFE, Transformer–RFE, RF, MOINER, and MOGONET. Features selected by more than one method are highlighted in bold. Predictive performance of the top five feature in a panel including disease classification accuracy (Accuracy) area under the receiver operating characteristics (AUC).

	SelectKBest	SVM-RFE	Transformer–RFE	RF	MOINER	MOGONET
**Multi-Omics Features**	**P80748**	**P01009**	**P01009**	**P80748**	P01042	**P0DOX5**
	**P0DOX8**	**P80748**	**P02656**	P04275	**P01009 (N-Gly)**	**P0DOX8**
	**P01009 (EnP)**	Q53H89	**heptacosanoic acid**	**P01009**	**heptacosanoic acid**	**P02656**
	**P01009 (N-Gly)**	**P0DOX8**	PC(18:0/20:0)	P0DOX2	P05154	A0A075B6R2
	**P0DOX5**	Q8WZ75	Q06033	O75882	P02768	**P01009**
**Accuracy**	100%	97.5%	97.5%	100%	87.5%	97.5%
**AUC**	1.00	1.00	1.00	1.00	0.938	0.988

**Table 5:** Number of significant features in each targeted omics dataset using Student’s *t*-test.

Omics Dataset	No. of Features	No. of Features (*p* < 0.05)
Metabolomics	53	5
Proteomics	101	43
Glycomics	82	8

**Table 6:** Top five-ranked features selected from the targeted dataset by six methods. Features selected by more than one method are highlighted in bold. Classification accuracy (Accuracy) and AUC values are calculated by using the top five features in a panel for disease classification via a logistic regression model and a five-fold cross-validation.

	SelectKBest	SVM-RFE	Transformer–RFE	RF	MOINER	MOGONET
**Multi-Omics Features**	**O75636**	**P03952**	tyramine	P01023	**isoleucine**	threitol
	**P03952**	**P05156**	**P01876**	**leucine**	P02652	ethanolamine
	**Q6EMK4**	**O75636**	P02774	**O75636**	**leucine**	sorbose
	P22891	6-hydroxy caproic acid	**P05156**	**Q6EMK4**	43211	**leucine**
	**P05156**	**P01876**	25000	**isoleucine**	P22891	creatinine
**Accuracy**	77.2%	75.0%	77.2%	72.8%	70.0%	68.0%
**AUC**	0.848	0.825	0.798	0.853	0.756	0.741

## Data Availability

The raw data supporting the conclusions of this article will be made available by the authors on request.
